# Pneumonia

**Published:** 1855-11

**Authors:** 


Lecture on Pneumonia. By M. Trousseau.—In No. 32 is a young girl who has suffered from pneumonia for six days. The disease commenced in the ordinary manner, with shivering, vomiting, pain in the side, fever, and characteristic expectoration. On examination a very extensive pneumonia was detected, occupying the apex of the left lung, descending in front and laterally to almost its base, leaving only the lower and posterior part of the organ in a sound state. The pulse was very frequent, and of very considerable force and fullness, when compared with the delicate constitution of the patient. To these symptoms were added labored respiration, extreme anxiety, and violent præcordial pain. These last symptoms led M. Trousseau to ask himself whether the pericardium had not become the seat of inflammation; for it is by no means rare to find a phlegmasia passing from the lung to the heart, and reciprocally, in consequence of the numerous anatomico-physiological ties that unite the two vicera, the coincident pericarditis being perhaps overlooked, owing to attention being conclusively fixed on the affection of the organ of respiration.

As in this patient, the entire absence of all physical signs proved the non-existence of pericarditis. The præcordial pain must be considered as dpending upon intercostal neuritis, shown by M. Beau to prevail in inflammation of the parietal pleura—a neuritis which, although affecting the trunk of the nerve, produces pain at its periphery, where the nervous filaments terminate. Thus, a phlegmasia, affecting a portion of the pleura near the vertebral column, induces pain at the anterior part of the chest. So, too, in a pleurisy of the basc we find the pain in the hypochondrium, and in that of the apex it has its seat, by reason of the intercostal nerves, in the præcordial region. This was the case with the patient, in whom the pneumonia was accompanied by a pleurisy of the apex.

The prognosis was serious for several reasons, such as the seat of the affection, its extent, and the violence of the febrile reaction. Bleeding seemed contra-indicated by the constitutional debility of the patient, and it was deemed prudent to depend upon *kermes mineral*, a preparation of antimony of powerful action, far more manageable than tartar emetic, while it possesses nearly all its physiological and therapeutical attributes, causing (like it) vomiting, and at an early stage of its administration, and, somewhat later, diarrhœa. It is very much preferable to administer antimony in the pilular form. When it has been given for several days in succession in the fluid form, it produces in the throat and œsophagus a diffused, special irritation, followed by pustules, analogous to those brought out on the skin by the use of the ointment. Kermes mineral produces this effect as well as tartar emetic, though less speedily, and in a less degree. Laennec was aware of the existence of these pustules, and regarded them as a sign of antimonial saturation, analogous to mercurial saturation. That this was an error is shown by giving the antimony in the form of pills, when pustulation never occurs. Another advantage they possess is, that even when given in double or triple doses, they will not so easily induce vomiting or diarrhœa as does the fluid. Thus, this patient took, during the first two days, fifteen grains, divided into ten pills, one every hour, without any inconvenience.

Some have attributed the therapeutical action of antimonials to an intestinal revulsion. Such was the opinion of Broussias, declaring that all Laennec did was to apply a blister to the intestines; and that admirable clinical observer, Chomel, entertained similar views. Laennec, on the other hand, believed that the remedy was absorbed, and accorded to it in the contra-stimulant virtue claimed for it by the school of Rasori. We believe we may simplify the problem by suppressing the vomiting and purging, which furnish, in appearance, some grounds to the partisans of the revulsion theory. Thus, in this patient, with the most complete tolerance of the remedy, the pneumonia remaining locally the same, you find the heat of skin has disappeared, and there is scarcely any frequency of pulse remaining. The antimony has vanquished the reactional phenomena. Absorbed, it has acted upon the nervous system, and through this upon the center of circulation. In proceeding thus we interrupt the chain established, through the intermedium of the nerves, between the circulatory centers and the organ affected, so that this last remains with its lesion isolated in the chest, as you may ascertain. But the fever and the general phenomena that accompanied it have disappeared, and this is an immense result; for, while we must not neglect the local condition, it is far more important to note the indirect action which this condition exerts upon the economy. It was against this action we directed our contra-stimulant, and we have been able to dissipate the excitement, the kind of titillation of the nervous system produced by the local lesion which remains now alone, deprived of the reactions which at first accompanied it. We must, in medicine, bear in mind other things than local conditions and suffering organs, regarding man as a whole, and the manner in which he is impressed by these. It is to the general condition alone that medication is oftenest addressed, and that even by the partisans of localization, who are far less exclusive than they believe, boldly opening a vein in pneumonia without considering whether this practice is in accordance with their theoretical views.

The kermes will in this case be continued for some days and then suspended, or we shall find the very pustulation produced in the stomach and intestines that we sought to avoid in the upper part of the canal. In a patient in one of the wards convalescence has been delayed by an enteritis thus artificially induced. As a general rule, medication is continued too long in internal affections, perhaps because we are not able to form an exact idea of the condition of the parts. We should rather imitate the conduct of the surgeon, who, after having procured a free issue for the pus of a phlegmon, leaves to nature the task of resolving the peripheric engorgement and remaining inaflmmation. The proper time for staying the hand, is when resolution has freely commenced. Thus, in pneumonia, as soon as the general phenomena have disappeared, and we hear the local signs of returning normal respiration, we must suspend medicines and have recourse to food.

